# Research on the Time Drift Stability of Differential Inductive Displacement Sensors with Frequency Output

**DOI:** 10.3390/s22166234

**Published:** 2022-08-19

**Authors:** Xiaolong Lu, Guiyun Tian, Zongwen Wang, Wentao Li, Dehua Yang, Haoran Li, You Wang, Jijun Ni, Yong Zhang

**Affiliations:** 1 School of Mechanical Engineering, Sichuan University, Chengdu 610065, China; 2 School of Engineering, Newcastle University, Newcastle upon Tyne NE1 7RU, UK; 3 School of Automation Engineering, University of Electronic Science and Technology of China, Chengdu 611731, China; 4 College of Automation Engineering, Nanjing University of Aeronautics and Astronautics, Nanjing 211106, China; 5 National Astronomical Observatories, Nanjing Institute of Astronomical Optics and Technology, Chinese Academy of Sciences, Nanjing 210042, China

**Keywords:** differential inductive frequency output displacement sensor, inductance digitization, LDC, time drift, stability

## Abstract

An edge displacement sensor is one of the key technologies for building large segmented mirror astronomical optical telescopes. A digital interface is one novel approach for sensor technologies, digital transformation and the Internet of Things (IoT) in particular. Frequency output sensors and inductance-to-digital converter (LDC) demonstrated significant advantages in comparison with conventional sensors with analog-to-digital converter (ADC) interfaces. In order for the differential inductive frequency output displacement (DIFOD) sensor to meet the high-stability requirements of segmented mirror astronomical telescopes, it is important to understand the factors for time drift of the sensor. This paper focuses on the investigation of key factors of sensor structure and material, signal conditioning and interface, and fixtures for time drift to permanently installed applications. First, the measurement principle and probe structural characteristics of the sensor are analyzed. Then, two kinds of signal conditioning and digitalization methods using resonance circuits and LDC chips are implemented and compared. Finally, the time drift stability experiments are performed on the sensors with different signal conditioning methods and fixtures under controlled temperature. Experimental results show that the magnetic shield ring effectively improves the sensitivity and quality factor of the sensors, the time drift stability of the sensor using the signal conditioning based on resonance circuits is better than that of the sensors using LDC chips, and the root mean square (RMS) of the sensor time drift meets the requirement of 0.01 μm/24 h. This study will help further development of high-stability of frequency output sensors and IoT-based systems for scaled-up applications in the future.

## 1. Introduction

The emergence of segmented mirror active optics technology has made it possible to build larger aperture optical or infrared astronomical telescopes, for example, the Large Sky Area Multi-Object Fiber Spectroscopic Telescope (LAMOST), which is China’s first astronomical national major science and technology infrastructure [1]. To improve the diffraction-limited image quality of the telescope for high resolution and high-efficiency astronomical observation, the positioning accuracy of all sub-mirrors from the segmented telescope primary mirror is required to meet the piston error requirements of several tens of nanometers RMS for active optics co-phase via permanently installed monitoring. Therefore, piston errors, known as position differences in the normal direction between neighboring sub-mirrors, need accurate measurements by edge displacement sensors, which are the important key technology for co-phase maintenance monitoring of segmented mirror active optical systems and corrections in real time by micro-displacement actuators [2].

At present, the edge displacement sensors used in large-scale splicing telescopes mainly include capacitive [3,4,5], inductive [6] and eddy current sensors [7]. These three types of sensors have their own advantages and disadvantages, among which the advantages of inductive sensors are prominent. They have the characteristics of low requirements in the working environment, high resolution, and good stability [8]. However, the signal conditioning methods for inductive sensing mainly involve an alternating current bridge circuit or inductive coupling via transformer, and the output signal is a voltage signal, which requires a complicated ADC circuit to convert it into a digital signal.

The DIFOD sensor is a frequency output type inductive displacement sensor, which has a digital interface through a resonant circuit without ADC. It was first proposed and invented by professors Zhang and Tian in 1988 [9]. Using multi-differential design (structure, signal conditioning circuit), its performance indicators have reached the sub-micron level, which fully meets the requirements of industrial automation, and has been widely adopted in various equipment developed by Sichuan University (previously known as Chengdu University of Science and Technology) for different industrial fields, e.g., metrology instrument, automobile and pipeline inspection [10,11].

However, when it is used in astronomical telescopes, its current performance has been unable to meet the requirements of co-phasing in the harsh environment of observatory conditions, especially its stability and sensitivity to temperature and humidity fluctuations. To make the DIFOD sensor meet the high-stability requirements of the segmented mirror telescope, this paper further investigates how their components affect their performance and new miniaturization via IC chips.

To some extent, the stability of the sensor is affected by temperature drift and time drift. Temperature drift is usually associated with changes in temperature or electronic components. Time drift is usually associated with the aging of transducer or electronic components. The stability of the sensor can be effectively improved by drift compensation. At present, drift compensation methods reported in the literature can be categorized into hardware compensation methods and software methods. The former is mainly based on material selection, structure optimization and a signal conditioning circuit designed to compensate for the drift [12,13,14,15]. The latter compensates for drift through various software algorithms [16,17,18]. These studies also show that drift compensation are carried out based on the influence rule or pattern of sensor drift with time and temperature variation, which is a time-consuming process. Before compensating for temperature drift, the time drift of the sensor system at a controlled temperature first needs to be investigated. Temperature drift and compensation can be further studied based on the findings of subsequent time drifts. This paper studies the influences of structure and material, signal conditioning and interface and fixture on the time drift or stability of DIFOD sensors under controlled temperature.

Industry 4.0 requires digital transformation and digital interface by introducing the IoT and information and communication technology (ICT), which serve as an interface between the digital and physical world [19]. The frequency output sensor has recently received a lot of attention because it does not require a complicated ADC circuit, can be inline readout [20,21] and has the potential and capacity to be integrated into any complex system [22,23]. Sensor data can be uploaded to the cloud via Wi-Fi or narrowband Internet of Things (NB-IoT) for structural health monitoring (SHM), such as a segmented telescope primary mirror [24]. Texas Instruments released the LDC 1000 in 2013, which enables high-performance and reliable inductive sensing at a very low cost and power consumption [25]. This was a major breakthrough in the field of sensors. The LDC chips use the same measurement principle as the DIFOD sensor, including frequency measurement [26]. It has not only been applied in consumer and industrial products but has also increasingly received attention in the field of non-destructive testing [27,28,29]. The LDC chips will help to further improve the stability, reliability, and miniaturization of the frequency output sensor, as well as develop an IC-based DIFOD sensor for temperature compensation and IoT-based applications. This paper investigates the time drift of two DIFOD sensors using two different signal conditioning methods: a resonance circuit and LDC chips. This is important for application in segmented mirror astronomical optical or infrared telescopes.

In this paper, DIFOD sensors were designed using resonance circuits and LDC chips, respectively. Then, different experiments were carried out to study the influence of the sensor structure and material, the signal conditioning circuit, and the fixture on the time drift of DIFOD sensors under a controlled temperature. The rest of this paper is organized as follows. Section 2 describes the measurement principle and structure of the DIFOD sensor and compares two kinds of signal conditioning and digitalization methods. The experimental setup and results analysis are presented and discussed in Section 3. Finally, Section 4 concludes and outlines future work.

## 2. Measurement and Different Operations for Digitalization

### 2.1. DIFOD Sensor Measurement Principle

**Figure 1:**
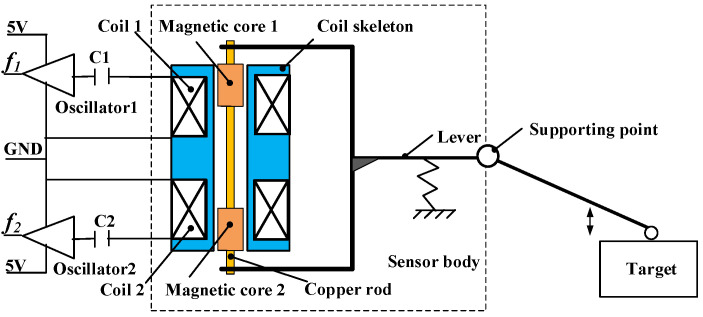
Working principle of the lever-type DIFOD sensor.

A DIFOD sensor is a kind of inductive displacement sensor based on the frequency output principle, using a multi-differential method to improve sensor performance. Figure 1 shows the working principle diagram of the lever-type DIFOD sensor. Two coils wound around a cylindrical coil skeleton and two magnetic cores are fixed on a copper rod. The two magnetic cores are separated by a certain distance. The copper rod is fixed on a U-shaped end of the lever. When the displacement between the probe and the target changes, the magnetic cores move up and down in the coil skeleton under the drive of the lever. Coil 1 and Coil 2 are part of oscillator 1 and oscillator 2, respectively. The changes in inductance in the coil cause the frequency of the oscillators to change in opposite directions. The frequency values of the two oscillators are measured using a frequency measurement module, and the difference between them is the measured value of the DIFOD sensor. The sensor data can either be transmitted to a computer or a microcontroller unit (MCU) through the communication bus for stand-alone measurement or uploaded to the cloud via Wi-Fi or NB-IoT for structural health monitoring.

**Figure 2:**
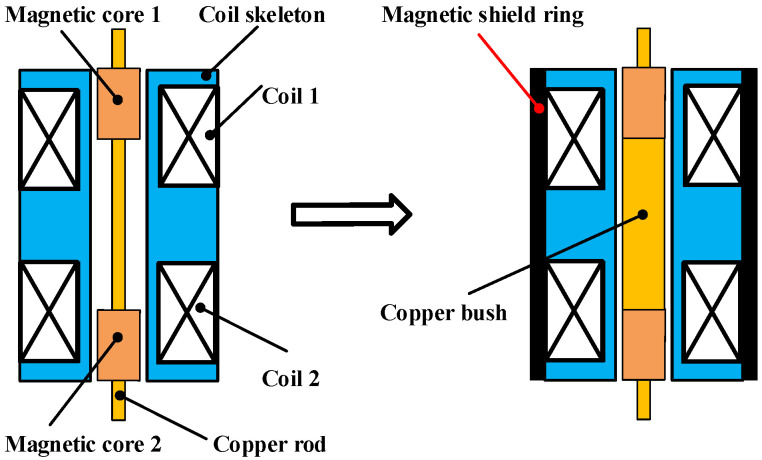
Structure optimization of DIFOD sensor.

To further improve the performance of the DIFOD sensor to meet the requirements of the segmented mirror telescope, magnetic shield ring and copper bush are used in the latest sensors to reduce the magnetic traction between coils 1 and 2, as shown in Figure 2. Finite element simulation (FEM) software COMSOL Multiphysics 5.6 was employed to analyse the new structure. Taking into account the cylindrical symmetry of the coil skeleton, only half of the longitudinal section of the sensor is modeled according to the model parameters in Table 1.

**Figure 3:**
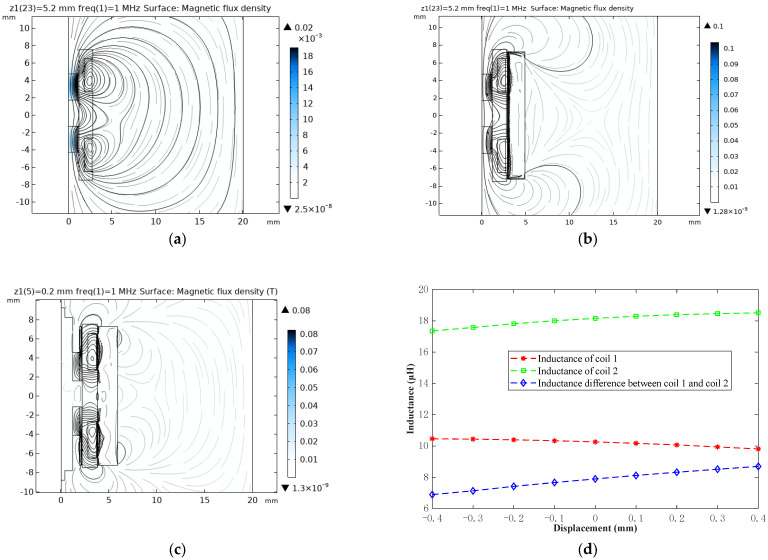
Finite element simulation results of the DIFOD sensor: (**a**) Magnetic flux density distribution (not using a magnetic shield ring and the copper bush); (**b**) Magnetic flux density distribution (only using magnetic shield ring); (**c**) Magnetic flux density distribution (using magnetic shield ring and copper bush); (**d**) The relationship curve of the inductance of the differential coil with the displacement.

Figure 3 illustrates the results of the simulation analysis. The magnetic flux density distribution without the use of a magnetic shield ring and copper bush is shown in Figure 3a, where the magnetic field lines of the magnetic field generated by coil 1 and coil 2 are closed curves, and the two magnetic fields pull each other. When the two coils are put into the magnetic shield ring, most of the magnetic field lines are cut off, and there are still magnetic field lines passing through the center of the coils, as shown in Figure 3b. As presented in Figure 3c, the magnetic field lines generated by coil 1 and coil 2 are effectively cut off by the magnetic shield ring and the copper bush, and the magnetic traction between coils 1 and 2 is effectively reduced. The sensitivity and quality factors of the sensor have been improved. Figure 3d shows that the inductance value and the difference of the two coils change linearly with the change in the magnetic core displacement. The sensor stability could be improved by separately compensating for the two coils’ drift. The refined differential structure with thermally stable materials will be compared and evaluated using different signal conditionings for frequency outputs.

### 2.2. Differential Operation Principles and Signal Conditioning

Two different kinds of signal conditioning, using traditional LC oscillation (resonance) circuits and LDC chips, are used for inductive probes for frequency outputs.

#### 2.2.1. Signal Conditioning and Digitalization Based on Discrete Devices and PCB Boards

**Figure 4:**
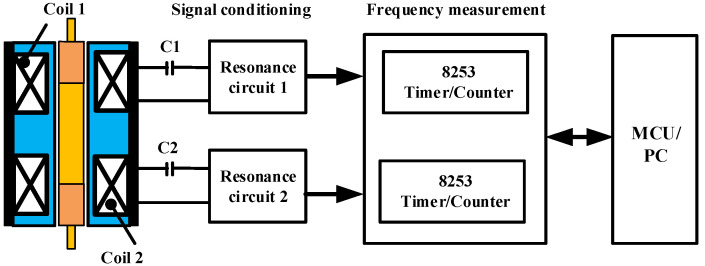
Block diagram of a signal conditioning circuit based on discrete devices.

Figure 4 is a block diagram of the signal conditioning and digitalization module of the DIFOD sensor, which has been used and optimized since this kind of sensor was invented in 1988 [9]. The module is composed of two resonance circuits based on an oscillator, a shaping amplifier and two channel frequency measurement circuits. The capacitors C1 and C2 form two LC oscillators with sensor coils 1 and 2, respectively. The signal conditioning circuit outputs a sine-wave signal with a frequency range of 1.0–2.0 MHz, and uses the two channel frequency measurement module to complete the counting of the measured signal. The measurement process can be controlled by an MCU or a personal computer (PC).

The frequency measurement circuit is composed of a programmable counter or a timer IC 8253. The chip consists of three independent 16 bit counters. While counter 0 is used to count and divide the frequency (*f_x_*) from the sensor, counter 1 and counter 2 form one 32 bit counter to count the high-frequency pulse signal (*f_φ_*) provided by the external crystal oscillator. The output of counter 0 is used as a gate control signal to control counter 1. The initial value *N_x_* of counter 0 is used to determine counting time using down counting. The higher the resolution of the sensor, the longer the counting time. This can be seen in reference [11]:

(1) $$\frac{N_{x}}{f_{x}}=\frac{N_{\phi }}{f_{\phi }}\Rightarrow f_{x}=\frac{N_{x}}{N_{\phi }}f_{\phi }$$

where *N_x_* is the initial value of counter 0, *f_x_* is the frequency of the sensor, *N_φ_* is the count value of the 32-bit counter, and *f_φ_* is the frequency of the external crystal oscillator.

As discussed in references [10,11], multi-counters can improve measurement resolution or measurement dynamic responses. The re-investigation of the approach will determine whether it meets the requirements of LAMOST in terms of time drift and in comparison with recent developments of LDC for frequency output.

#### 2.2.2. Signal Conditioning and Digitalization Based on LDC Chips

**Figure 5:**
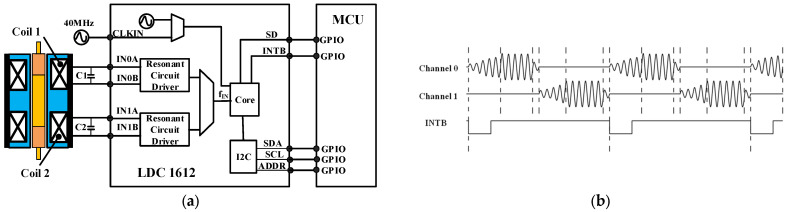
Signal conditioning circuit based on LDC 1612; (**a**) Functional block diagram of the signal conditioning circuit; (**b**) Control timing.

The LDC 1612 is an inductance-to-digital converter supplied by Texas Instruments. The device can measure the oscillation frequency of multiple LC resonators and output a digital value that is proportional to the frequency with a 28-bit measurement resolution [26]. As shown in Figure 5a, the LDC 1612 is composed of two front-end resonant circuit drivers and a core that measures and digitizes the sensor frequency. The I2C interface is used to support the device configuration and transmit the digitized frequency values to the MCU. With the register of FREF_DIVIDERx and FIN_DIVIDERx set to 1, the sensor frequency can be calculated from

(2) $$f_{sensor}=\frac{DATAx\times f_{REFx}}{2^{28}}$$

where *f_sensor_* is the sensor frequency, the *f_REF__x_* is the reference clock of Channel x, and *DATA_x_* is the count value of Channel x.

The resolution of the sensor can be changed by adjusting the value of the *RCOUNT_x_* register that controls the sensor conversion time. The higher the resolution of the sensor, the longer the conversion time. Unlike the signal conditioning circuit based on discrete devices, LDC 1612 provides flexibility in channel sampling. When operated in multi-channel mode, the LDC sequentially samples the selected channels, as shown in Figure 5b. When the data conversions of channel 0 and channel 1 are completed, the INTB pin is set to a low level. This will trigger the MCU interrupt to read the conversion results through the I2C.

As illustrated in Figure 5b, sensor activation at different drive currents can provide a different transient response and dynamic responding time for the sensor oscillation amplitude to be stabilized. It can be applied to transient responses and different inductance responses, which can be linked to the dynamic responses of different equivalent models of inductance and eddy current sensors [30]. Compared with the signal conditioning and digitalization circuit based on discrete devices, LDC 1612 integrates the resonance circuit, frequency measuring and I2C bus. The multi-channel package of the LDC enables the system design to be more flexible. The LDC chips not only help to improve the stability and reliability of DIFOD sensors but also contribute to the miniaturization and intelligence of DIFOD sensors and their future application in the IoT. Therefore, comparing the above two signal conditioning and digitalization methods is important to improve the performance of the DIFOD sensor and to develop an IC-based DIFOD sensor for temperature compensation and IoT-based applications.

## 3. Experimental Rig and Testing

The experimental setup and sensor probe configuration are presented and discussed in this section. The time drift measurement of the DIFOD sensors, using different signal conditioning, digital interfaces, and fixtures, was carried out under controlled temperatures in this section. It will identify the time drift pattern under different signal conditions and test rigs, including fixtures.

### 3.1. Experimental Setup and Different Displacement Sensors

**Figure 6:**
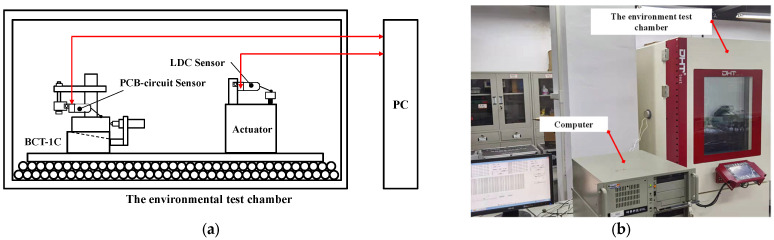
Experimental Section setup: (**a**) Experimental Section setup block diagram; (**b**) Picture of experiment setup.

As shown in Figure 6, including the experimental setup block diagram and picture, an experimental setup for the sensor time drift assessment was designed. The environmental test chamber produced by Duohe Testing Equipment Co., Ltd. (Shanghai, China), illustrated in Figure 6b, is used to provide temperature control for the time drift evaluation experiments. The computer is employed to acquire and analyse sensor data. A temperature of 20 °C is controlled for time drift measurement in the studies below.

**Figure 7:**
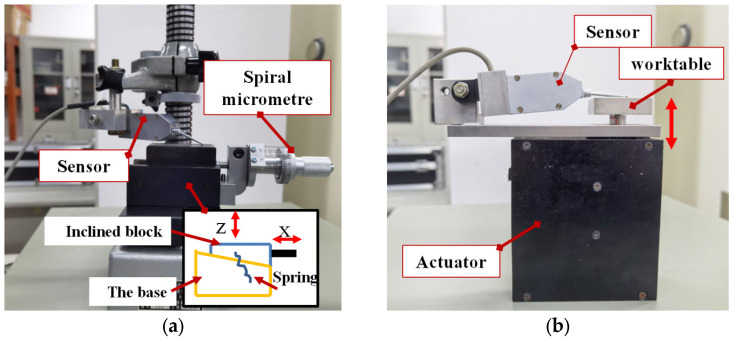
Sensor fixtures for time drift tests of DIFOD sensors: (**a**) BCT-1C or BCT-5C fixtures; (**b**) Micro-displacement actuator.

A micro-displacement measuring mount, BCT-5C, produced by Zhongyuan Measuring Instrument Co., Ltd., is used to calibrate the DIFOD sensors. The device can produce a precise amount of displacement. The BCT-5C and BCT-1C are the same types of equipment, producing different micro-distance resolutions of 0.2 μm and 1.0 μm, as illustrated in Table 2. As shown in Figure 7a, the device is mainly composed of a spiral micrometer, inclined block, and base. The spiral micrometer drives the inclined block to move along the inclined surface of the base. When the spiral micrometer moves horizontally by the displacement Δ*s*, the inclined block rises by Δ*h*, it is:

(3) Δh=Δstanα

where α is the inclination angle of the inclined block for the micro-displacement measuring mount separately. The α of BCT-1C and BCT-5C are 1:10 and 1:50, respectively.

To calibrate the sensors and study the influence of different fixtures on the stability or time drift assessment of the sensor, as seen in Figure 7a, the two fixtures have different purposes. In terms of displacement resolution, the BCT-5C and BCT-1C can provide different resolutions for calibration with complex geometry. In addition to the BCT-5C and BCT-1C fixtures manually driven by a precision screw, a precision micro-displacement actuator driven by a stepper motor with a planetary reducer is employed to test the sensors, as shown in Figure 7b, where the actuator has a compact geometrical configuration, as discussed in [2]. Compared with the micro-displacement actuator, the BCT-1C and BCT-5C are complex in the structure. In the future, the micro-displacement actuator is designed to form a closed-loop co-phase maintenance system with DIFOD sensors and optical mirrors. In this paper, the BCT-5C fixture is used to calibrate the displacement sensors with a resolution of 0.01 μm and measurement ranges of ±200 μm; the micro-displacement actuator is also used to study the influence of different fixtures on sensor time drift. The fixtures with tested sensors are placed on layered table tennis balls to minimize the influence of environmental vibration, as shown in Table 3.

### 3.2. Sensors Probe Structure and Materials

**Figure 8:**
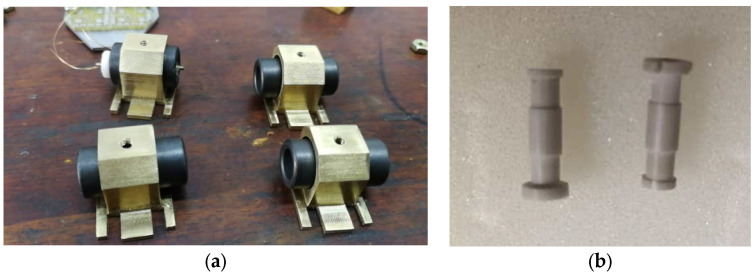
Sensor coil and skeleton: (**a**) Magnetic shield ring; (**b**) Coil skeleton.

Through the comparison and analysis of several commonly used sensor coil skeleton materials, such as polytetrafluoroethylene, plexiglass and ceramics, and aluminum nitride ceramic materials, with a relatively small thermal expansion coefficient, are chosen to manufacture the coil skeleton, as shown in Figure 8b. Based on the discussion of FEM simulation results in Section 2.1, a magnetic shield ring is used to improve the sensitivity and quality factor of the sensor, and a copper bush is used to reduce the magnetic traction between the coils, as shown in Figure 8a. In addition, C0G (NP0) ceramic capacitors with excellent temperature stability are used in signal conditioning (oscillator) circuits. Using the proposed sensor probe’ differential configuration, four lever-type DIFOD sensors were developed. Two of the sensors used the signal conditioning method based on discrete devices, namely PCB circuit sensors 1 and 2. The other two sensors employed the signal conditioning method based on LDC chips, namely LDC based sensors 1 and 2. The inductance of the two coils are about 43 μH and 56 μH, the capacity of the capacitors (C1 and C2) in the resonant circuit are 200 pF, and the resonant frequency is about 1.72 MHz and 1.53 MHz, respectively. Table 3 illustrates the signal conditioning circuits, level-type displacement sensor probes and experimental setups on table tennis balls in the test chamber. The four sensor probes use the same mechanical structure with the same parameters. The sensor signal conditioning circuits are installed in the box at the end of each sensor probe. These sensors need to be fixed on the fixtures first and then placed in the environmental test chamber for time drift stability evaluation.

### 3.3. Comparison Experiment of Different Signal Conditioning Circuits

In this paper, the time drift is studied under the same sensor coils, differential configuration and materials as discussed in Section 3.1. Four DIFOD sensors, using two different signal conditioning circuits, as mentioned above, are tested for time drift under a controlled temperature. The temperature drift and compensation will be reported in the future.

#### 3.3.1. Time Drift or Stability under Controlled Temperatures

**Figure 9:**
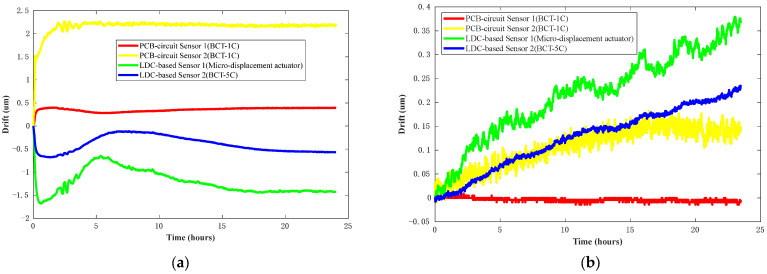
Test results for long-term stability or time drift under controlled temperatures: (**a**) Time drift within 24 h after installation; (**b**) Time drift after 24 h of stability.

To compare the time drift of the DIFOD sensors using different signal conditioning circuits, the four sensors are first calibrated with the BCT-5C. The measurement range of the sensor is 0–300 μm. Then, the four sensors are installed on the BCT-1C, BCT-5C, and micro-displacement actuator and these are placed together in the environmental experimental box. The displacement values of the four sensors are 150 μm by adjusting the fixtures. The temperature of the environmental experiment box is controlled at 20 °C. The sensor data are recorded at every minute. The entire experiment requires 24 h to stabilize. Figure 9 presents the experimental data for the stability assessment of the four sensors. Figure 9a illustrates some directional drift within 24 h after installation; Figure 9b illustrates the time drift of the four sensors after stabilization of 24 h.

As shown in Figure 9a, the PCB circuit sensor undergoes stabilization faster than the LDC based sensor. The reason for this is mainly the different working modes of the two signal conditioning circuits. The PCB circuit sensor samples the two channel frequencies simultaneously, continuously providing an excitation current to the two coils. Meanwhile, the LDC based sensor alternately samples the frequencies of the two channels and intermittently provides an excitation current to the two coils. The coils of the PCB circuit sensor take less time to reach thermal stability than the coils of the LDC based sensor.

In addition, as can be seen from Figure 9a that the four sensor drift curves oscillate at the same time at the beginning of the experiment. The direction of the oscillation is the same, but the amplitude is different. The main reason for this is that the oscillation is caused by the environmental vibration of fixtures during the time drift tests. The fixed state of the sensor, the fixture, and the placement of the fixture lead to different oscillation amplitudes of the drift curves of different sensors. As shown in Figure 9b, the two PCB circuit sensors were stabilized after 24 h, while their drift curves are different. The RMS of the time drift of PCB circuit sensors 1 and 2 is 0.0055 μm and 0.1200 μm, respectively. The two LDC based sensors are still not stabilized, but their drift curves have a similar pattern. The main reason is that the signal conditioning circuit of the PCB circuit sensor is composed of discrete devices. It is difficult to guarantee that the temperature coefficient of each discrete device is the same in a scaled-up application. Therefore, there will be a big difference in the drift curves of the two PCB circuit sensors. In contrast, the signal conditioning and digitalization of the LDC based sensors are completed by one LDC1612 chip, which has good consistency.

#### 3.3.2. Comparison of Experiments with Different Sensor Fixtures and Different Sensors

To study the influence of different sensor fixtures on the time drift stability of the DIFOD sensor, PCB circuit sensors 1 and 2 are fixed on the BCT-1C and micro-displacement actuator, respectively, and then placed together into the environmental experimental box. The displacement values of the four sensors are 150 μm by adjusting the fixtures. The temperature of the environmental experiment box is controlled at 20 °C. The sensor data are recorded once every minute. After 48 h, the two sensors’ fixtures are exchanged and the time drift experiment is repeated.

**Figure 10:**
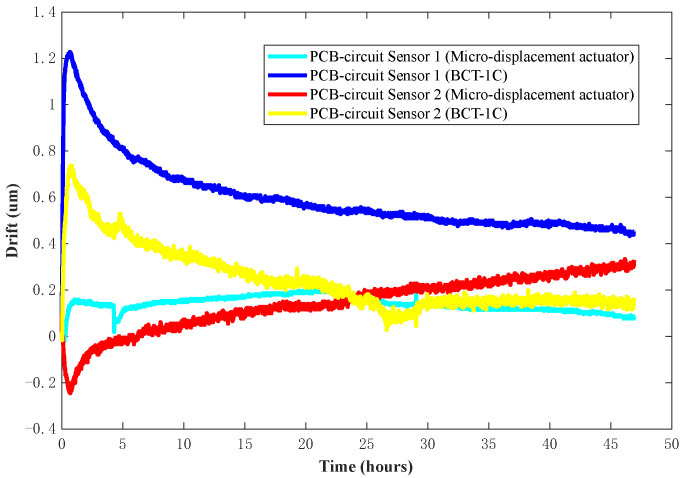
Test results of time drift stability with different fixtures within 48 h after installation.

Figure 10 shows the time drift results of PCB circuit sensors 1 and 2 fixed on different fixtures within 48 h after installation. The experimental results show that the sensor fixed on the BCT-1C takes longer to be stabilized than the sensor fixed on the micro-displacement actuator. In the first 10 h, time drifts from sensors 1 and 2 on BCT-1C are 0.6 and 0.3 μm, while time drifts from sensors 1 and 2 on micro-displacement actuator fixture are 0.2 and 0.3 μm. In the remaining 38 h after the first 10 h, time drifts from sensors 1 and 2 on BCT-1C are 0.2 and 0.4 μm, while time drifts from sensors 1 and 2 on micro-displacement actuator fixture are 0.2 and 0.2 μm with clear stablisation as shown in Figure 10. The main reason is that the structure of the micro-displacement actuator is simpler than that of the BCT-1C (as shown in Figure 7), and its deformation is smaller.

## 4. Conclusions and Future Work

In this paper, two kinds of differential frequency output inductive displacement sensors, using different digitalization operation principles and signal modulation circuits, are proposed and evaluated. The time drift stability evaluation experiments on the sensors were carried out with different structures, materials, signal conditioning circuits, and test fixtures for the comparison of different signal conditioning via discrete devices and LDC chips. The following conclusions are drawn from this study:

(1) The magnetic field of the two coils is well isolated using the magnetic shield ring and the copper bush. They effectively reduce the traction between the magnetic fields of the two coils and improve the sensitivity and quality factors of the sensor. The probe with improved probe structure and material, using a signal conditioning circuit based on a discrete device, meets the time drift RMS requirement of 0.01 μm/24 h following 24 h after installation.
(2) The time drift stability of the sensor systems, using a signal conditioning circuit based on a discrete device, is better than that of the sensor using LDC chips due to the different sensor coil excitation methods. Sensors using LDC chips can provide better consistency stability due to the chip integrating resonance circuit and frequency measurement module.
(3) The experimental fixture has an impact on the stability or time drift of the DIFOD sensor; the sensor fixed on the BCT-1C takes longer to be stabilized than the sensor fixed on the micro-displacement actuator due to fewer drift factors. This finding is helpful when choosing an experimental fixture for future temperature compensation.

The influence factors of time drifts and their mitigation methods via sensors’ structure and material, signal conditioning and digital interface provide an important base for future temperature drift compensation. Future research includes further research on time drift compensation methods based on artificial intelligence (AI) and LDC displacement sensors for time and temperature drift compensation and IoT-based applications.

## Figures and Tables

**Table 1:** Sensor structure design parameters.

Parts	Parameters	Value
Coil skeleton	Length (mm)	14.5
	Outer diameter (mm)	5.2
	material	Aluminum nitride ceramics
	Coil distance (mm)	4.0
Coil 1	Wire diameter (mm)	0.1
	Number of turns Material	90 Copper
Coil 2	Wire diameter (mm)	0.1
	Number of turns Material	120 Copper
Magnetic Cores 1 and 2	Core length (mm)	3.0
	Outer diameter (mm) Material	2.5 Ferrite core
Magnetic shield ring	Length (mm)	14.5
	Inner diameter (mm)	5.5
	Outer diameter (mm) Material	9.5 Nickel Zinc ferrite

**Table 2:** Micro-displacement measuring mounts.

Parameters	BCT-5C	BCT-1C
Displacement resolution	0.2 μm	1.0 μm
Measuring range	0~0.4 mm	0~2.0 mm
Measuring error	±0.12~±0.2 μm	±0.5~±3.0 μm
Slope of the inclined block	1:50	1:10

**Table 3:** Prototype of DIFOD sensors’ structure, signal conditioning devices and experimental setup.

Types of Displacement Sensors	Signal Conditioning (Oscillators) Circuits	Sensor Probes with Differential Structure	Experiment Setup in the Test Chamber
PCB circuit sensor	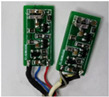	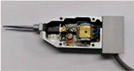	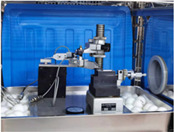
LDC-based sensor	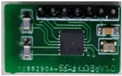	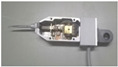	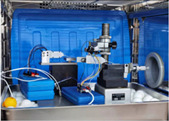

## Data Availability

The data presented in this study are available on request from the corresponding author.
